# An Artificial-Intelligence-Discovered Functional Ingredient, NRT_N0G5IJ, Derived from *Pisum sativum*, Decreases HbA1c in a Prediabetic Population

**DOI:** 10.3390/nu13051635

**Published:** 2021-05-13

**Authors:** Sweeny Chauhan, Alish Kerr, Brian Keogh, Stephanie Nolan, Rory Casey, Alessandro Adelfio, Niall Murphy, Aoife Doherty, Heidi Davis, Audrey M. Wall, Nora Khaldi

**Affiliations:** Nuritas Ltd., Joshua Dawson House, Dawson St, Dublin 2, Ireland; chauhan.sweeny@nuritas.com (S.C.); kerr.alish@Nuritas.com (A.K.); keogh.brian@nuritas.com (B.K.); nolan.stephanie@nuritas.com (S.N.); casey.rory@nuritas.com (R.C.); adelfio.alessandro@nuritas.com (A.A.); info@nuritas.com (N.M.); doherty.aoife@nuritas.com (A.D.); davis.heidi@Nuritas.com (H.D.); n.khaldi@Nuritas.com (N.K.)

**Keywords:** bioactive peptides, glucose uptake, functional ingredient, dietary efficacy

## Abstract

The prevalence of prediabetes is rapidly increasing, and this can lead to an increased risk for individuals to develop type 2 diabetes and associated diseases. Therefore, it is necessary to develop nutritional strategies to maintain healthy glucose levels and prevent glucose metabolism dysregulation in the general population. Functional ingredients offer great potential for the prevention of various health conditions, including blood glucose regulation, in a cost-effective manner. Using an artificial intelligence (AI) approach, a functional ingredient, NRT_N0G5IJ, was predicted and produced from *Pisum sativum* (pea) protein by hydrolysis and then validated. Treatment of human skeletal muscle cells with NRT_N0G5IJ significantly increased glucose uptake, indicating efficacy of this ingredient in vitro. When db/db diabetic mice were treated with NRT_N0G5IJ, we observed a significant reduction in glycated haemoglobin (HbA1c) levels and a concomitant benefit on fasting glucose. A pilot double-blinded, placebo controlled human trial in a population of healthy individuals with elevated HbA1c (5.6% to 6.4%) showed that HbA1c percentage was significantly reduced when NRT_N0G5IJ was supplemented in the diet over a 12-week period. Here, we provide evidence of an AI approach to discovery and demonstrate that a functional ingredient identified using this technology could be used as a supplement to maintain healthy glucose regulation.

## 1. Introduction

Type 2 diabetes mellitus (T2DM) is a chronic condition that affects glucose homeostasis within the body. It is characterised by a state of insulin resistance, and its prevention is impacted by the low conversion rate of exploratory research into efficacious therapies. The global incidence of type 2 diabetes is increasing, currently affecting 425 million people, and this is expected to increase by a further 204 million by 2045 [1]. Prediabetes is a condition indicated by higher-than-normal blood glucose levels due to increased insulin resistance or reduced pancreatic beta cell function [2,3]. The global incidence rate of prediabetes is also increasing rapidly, with 70% of prediabetics developing T2DM within their lifetime [4]. Long-term complications of developing diabetes carry massive health implications for patients, due to a number of associated health risks, as well as high costs for healthcare systems [5]. In 2010, concentration of glycated haemoglobin (HbA1c) was included as a diagnostic tool for prediabetes and subsequent development of T2DM [6]. HbA1c is considered a reliable biomarker of long-term glycaemic control as it reflects the exposure of red blood cells to glucose throughout their lifespan, roughly 2–3 months [7], although the guideline for testing is generally every 6 months [8]. Patients are considered prediabetic if their HbA1c levels are between 5.6% and 6.4% [9]. In a study conducted by the Heart Outcomes Prevention Evaluation (HOPE), results indicated that, for every 1% increase in HbA1C, there is a higher relative risk of 1.07 of cardiovascular outcomes [10]. Thus, clinically decreasing HbA1c levels has been indicated as a sensible approach to managing diabetes, supported by multiple clinical trials [11], and it also highlights the potential benefits for improving health by improving glycaemic control.

Currently, synthetic peptides are commonly used as treatments for T2DM with insulin analogues, and more recently incretin mimetics, acting as either analogues (e.g., GLP-1 and GIP analogues) or antagonists (e.g., GLP-1 receptor antagonists) of endogenous human hormones forming the core of a number of modern antidiabetic drugs [9]. However, a nutritional interventional approach to attenuate insulin resistance and prevent the development of T2DM at the stage of intermediate hyperglycaemia provides a potential alternative to prevent the health and economic costs associated with T2DM [12]. Diets supplemented with bioactive peptides can be used in an efficient and safe manner to promote health [13]. Yet discovering validated and characterised hydrolysates, containing an abundance of bioactive peptides, which can be taken chronically as nutritional interventions has proven difficult [14]. To overcome this hurdle, an EU framework was created by H2020 with the goal to support scientific innovations that solve EU-wide health challenges in a cost-effective manner and to take such innovations from the lab to the market [15]. As such, the creation of new natural, validated, functional ingredient interventions that target different disease areas in a risk reduced manner could address this issue. There are currently no validated nonpharmaceutical ingredients, with European Food Safety Authority approval, that target glucose management, despite its healthcare burden. Therefore, we utilised artificial intelligence (AI) to identify and produce an efficacious and cost-effective ingredient solution which aligns with the mission of H2020 of targeting this health challenge. AI, which includes machine learning approaches, has been successful in deciphering the complexity of natural source proteomes in order to elucidate noteworthy bioactive peptides. Recent discoveries include single bioactive peptides derived from the rice proteome with antiaging properties [16,17], while networks of bioactive peptides or hydrolysates have been successfully investigated for the prevention of inflammaging [18,19] and muscle atrophy [20]. An AI approach to discovery represents a safe and effective method to produce benefits, which have already shown a great effect of risk reduction by nutritional intervention [21], in a manner that is compliant-friendly.

The purpose of this study was to identify a peptide network, NRT_N0G5IJ, capable of modulating glycaemic control, using AI. The effect of NRT_N0G5IJ on glucose uptake in vitro in human skeletal muscle cells was assessed, followed by preclinical evaluation in a T2DM animal model. Finally, the effect of NRT_N0G5IJ was investigated in a double-blinded, placebo controlled clinical trial in a prediabetic population to assess the opportunity for a functional ingredient to prevent the development of T2DM. 

## 2. Materials and Methods

### 2.1. NRT_N0G5IJ Discovery and Production

To discover a functional ingredient with glucose-modulating activity, a predictive model was developed using a collection of neural networks. The predictive model utilised here was described by Casey et al. (2021), whereby a number of validated peptides were identified with blood glucose modulation activity [22]. Briefly, neural networks models based on stacks of recurrent and dense layers were trained in fold cross-validation using a dataset of glucose-regulating peptides that was built by combining data from literature with proprietary internal data established from mass spectrometry and in vitro investigations. Two sets of models were trained using different levels of internal redundancy for the training sequences. The ensemble of the best-performing models on the test sets was used as final predictive model for glucose-upregulating peptides. The predictor was used for in silico screening of several plant proteomes with a focus on the most abundant proteins for each plant. *Pisum sativum* resulted the top-ranked natural source by counts of peptides classified as active by our predictor in the area of glucose regulation and, therefore, was prioritised for in vitro testing.

NRT_N0G5IJ (PeptiForce^™^) was prepared using *P. sativum* as a source material and according to Kennedy et al. (2020) with some modifications [18]. In brief, *P. sativum* protein powder was homogenised in solution, and a sequential enzymatic hydrolysis with food-grade serine protease was carried out under monitoring and control of enzyme-specific conditions, such as temperature and pH value, which was adjusted using sodium hydroxide or hydrochloric acid. Following hydrolysis and enzyme inactivation at 85 °C for 10 min, the solution was spray-dried at 180 °C utilising a multistage Anhydro Spray Dryer at a facility accredited to FSC2200.

The study products were manufactured at Teagasc, Moorepark, under HACCP Food Quality Standards. No chemical additives were used, and the products were free of sulphites, sulphates, and aflatoxins. The source materials were produced in an ISO 22,000- and HAACP-compliant facility. The spray-dried powders were used for all assays and the murine model; they were prepared as follows: 150 mg of hydrolysate was weighed into a 50 mL tube. Then, 5 mL of water was added to the tube and vortexed until the powder was in solution. The tube was centrifuged at 4000 rpm for 20 min, and the supernatant collected into a new tube. The supernatant was filtered through 0.22 µM sterile filters and homogenised; protein content was measured using BCA.

### 2.2. Sample Preparation and Mass Spectrometry Analysis

Samples (100 μL) were desalted and concentrated using 30 kDa Spin-X UF centrifugal concentrators (Corning Inc., Lowell, MA, USA). Each flowthrough was then acidified in formic acid and cleaned of contaminants by solid phase extraction (SPE) on an Empore 96-well disc plate with C18-SD sorbent (3M, St. Paul, MN, USA). Eluates were lyophilised and resuspended in 0.1% TFA for analysis.

Samples were analysed by nano LC–MS/MS with a Waters NanoACQUITY HPLC system (Waters Corporation, Milford, MA, USA) interfaced to a ThermoFisher Q Exactive (ThermoFisher Scientific Inc., Canoga Park, CA, USA). Peptides were loaded on a trapping column and eluted over a 75 μm analytical column with a 1 h gradient at a flow rate of 350 nL·min^−1^. Both columns were packed with Luna C18 resin (Phenomenex, Torrance, CA, USA). The mass spectrometer was operated in data-dependent mode, with MS and MS/MS performed in the Orbitrap at 70,000 FWHM and 17,500 FWHM resolution, respectively. From the MS scan, the 15 most intense ions were selected for MS/MS.

### 2.3. Cell Culture and Assays

Human skeletal muscle cells (HSkMCs) were purchased from Cell Applications (San Diego, CA, USA) and cultured at 37 °C, 5% CO_2_ in HSkMC growth media (Cell Applications Inc., San Diego, CA, USA). HSkMCs were not used once they had undergone 10 passages.

### 2.4. Glucose Uptake Assay

HSkMCs were plated on sterile collagen-coated 96-well plates (1 × 10^4^ well^−1^) and allowed to adhere overnight at 37 °C, 5% CO_2_ in 100 µL of HSkMC growth media. The medium was changed to HSkMC differentiation medium (Promocell, Heidelberg, Germany) and cells were allowed to differentiate for 7 days, with the medium replaced every 2 days. Cells were serum-starved for 24 h prior to measurement of glucose uptake. Glucose uptake was measured using a glucose uptake assay kit (Abcam, Cambridge, MA, USA) as per the manufacturer’s instructions. Briefly, cells were rinsed three times in Dulbecco’s phosphate-buffered saline (DPBS; Lonza, Basel, Switzerland) and then starved of glucose by incubating with 100 µL of Krebs–Ringer–Phosphate–Hepes (KRPH) buffer for 40 min at 37 °C. Cells were treated with HSkMC basal medium containing NRT_N0G5IJ or insulin as indicated in the figure legend for 20 min, followed by incubation with 10 µL of 2-deoxyglucose (2-DG) for 20 min at 37 °C. Subsequently, cells were washed three times with PBS and lysed with 80 µL of extraction buffer, after which cell lysates underwent one freeze–thaw cycle before heating at 85 °C for 40 mins. Following cooling on ice for 5 min, lysates were neutralised by adding 10 µL of neutralisation buffer and then diluted with assay buffer to a total volume of 50 µL (5 µL of lysate + 45 µL of assay buffer). After an oxidation and amplification step, absorbance of the samples was measured at 412 nm with a microplate spectrophotometer (SpectraMax M3, Molecular Devices, Sunnyvale, CA, USA).

### 2.5. Mouse Model of Diabetes

This study was carried out by Melior Discovery, Exton, PA, USA. All animal procedures were carried out in accordance with Institutional Animal Care and Use (IACUC) guidelines in an Association for Assessment and Accreditation of Laboratory Animal Care International-accredited facility. Ethical approval was granted by the International Association of Religious Freedom (IARF #:MLR-101, 1 May 2018). Male 6–7 week old db/db mice (BKS.Cg-Dock7m+/+Leprdb/J) were sourced from Envigo and housed on a 12 h light/dark cycle with four mice per cage in a ventilated cage rack system with access to standard rodent diet and water available ad libitum. All experimental procedures were also approved by the onsite ethics committee. Mice were acclimatised for no less than 7 days, after which fasting glucose concentrations were determined to allow for randomisation.

Mice were dosed daily orally with vehicle or test compounds for a total of 43 days. The study consisted of four treatment groups (1) control vehicle—purified water; (2) rosiglitazone—15 mg/kg; (3) NRT_N0G5IJ—400mg/kg, and (4) unhydrolysed material (400 mg/kg). All mice received a volume of 5 mL/kg by oral gavage for each treatment.

Body weight measurements and fasting blood glucose were measured weekly. Average food and water intake were recorded twice per week. For determination of fasting glucose, mice were fasted overnight, and the blood glucose concentrations were measured using Glucocard Vital glucometers (Arkray, Minneaolis, MN, USA). Due to a staggering of blood draws on the animals, blood HbA1c concentrations were only measured at week 4 using the A1C Now Diagnostics Kit (PTS Diagnostics, Whitestown, IN, USA).

### 2.6. Double-Blind, Placebo-Controlled Trial of NRT_N0G5IJ in a Prediabetic Population

To characterise the effects of NRT_N0G5IJ in human, we carried out a randomised, double-blinded, placebo-controlled study where we administered NRT_N0G5IJ once daily over a 12 weeks period to prediabetic but otherwise healthy subjects (NCT03851666). The primary endpoint of this pilot trial was change in HbA1c concentrations in the blood. Secondary endpoints included postprandial glucose/insulin concentrations, BMI, weight, and fructosamine and fasting plasma glucose concentrations.

The trial was carried out at two sites, Stradini Hospital, Riga, Latvia and Adoria Health Centre, Riga, Latvia. Subjects were enrolled in accordance with the Declaration of Helsinki. Written, informed consent was obtained from all participants, and ethical approval was granted by the clinical research ethics committee of Stradini Hospital. The trial was carried out under the supervision of Prof Valdis Pirags (Stradini Hospital) and managed on behalf of Nuritas by Prof Christine Leiper (Onorach Clinical, Dundee, Scotland). All trial details can be viewed at Clinicaltrials.gov (trial protocol NCT03851666). Inclusion criteria were as follows:

- Provided written informed consent,
- Aged between 18 and 75 years, inclusive,
- HbA1c of >5.7% and <6.4% (38.8–47 mmol/mol),
- Non-smoker or an ex-smoker (10 years or more),
- BMI 20–35 kg/m²,
- Stable body weight (±5%) in the last 3 months (as self-reported by the subject),
- Willing to maintain existing dietary habits and physical activity levels throughout the trial period,
- Able to communicate well with the investigator, to understand and comply with the requirements of the study, and judged suitable for the study in the opinion of the investigator.

Exclusion criteria were as follows:

- Diagnosed diabetes with an HbA1c > 6.4% (47 mmol/mol),
- BMI less than 20 (underweight) or greater than 35 (morbidly obese),
- Significant acute or chronic coexisting illness such as cardiovascular disease, chronic kidney or liver disease, gastrointestinal disorder, endocrinological disorder, immunological disorder, metabolic disease, or any condition which contraindicates, in the investigator’s judgement, entry to the study,
- Consumption of more than the recommended alcohol guidelines i.e., >21 alcohol units/week for males and >14 units/week for females,
- Currently or recently (within 3 months of study entry) taking any medication, which, in the opinion of the investigator, could interfere with the outcome of the study, including insulin, acetylsalicylic acid, and thyroxine,
- Taking hypolipidemic agents and/or beta-blockers,
- Known allergy to any of the components of the test product,
- History of drug or alcohol abuse,
- Present or recent use (within 3 months of pre-screening) of dietary supplements that may affect the level of blood glucose,
- Low haemoglobin or haematocrit,
- Pregnant, lactating, or wishing to become pregnant during the study,
- Participation in a clinical trial with an investigational product within 90 days of pre-screening, or plans to participate in another study during the study period,
- History of noncompliance.

Briefly, eligible participants were healthy males and females aged 18–75 years with HbA1c > 5.7% and < 6.4% (38.8–47 mmol/mol) and BMI 20–35 kg/m^2^. Subject demographics are shown in Table 1.

Potential study subjects were recruited on the basis of the criteria above and assessed at a screening visit. At the first screening visit, subjects were also provided with a 4 days diet diary. The diary was completed by the subject for a total of 4 days before Visits 2 and 5. This diary ensured that a baseline of their typical dietary intake was recorded. The returned food diary was reviewed and analysed. Bloods were drawn at the screening visit and analysed, and, if they met inclusion criteria and still consented, an appointment was made to return to the study site within 3 weeks for the baseline visit. Subjects returned to the site fasting for at least 10 h. For blood draws, a cannula was placed in the subjects’ nondominant arm and pre-administration blood draws were carried out for determination of baseline values for glucose, insulin, fructosamine, and HbA1c concentrations. 

An oral glucose tolerance test (OGTT) was then performed as follows: a drink solution was prepared by dissolving 75 g of anhydrous glucose in a standardised volume of water and was consumed by subjects within 5 min. The time at which the drink was finished was recorded, and additional blood samples were drawn at 30, 60, 90, and 120 min after this time, from which glucose and insulin levels were obtained.

Subjects were randomly assigned to either the treatment (NRT_N0G5IJ), placebo (Avicel^®^ PH Microcrystalline Cellulose), or protein hydrolysate control (rice NPN) group. Rice NPN was produced as per Kennedy et al. (2020) [18]. All treatments were supplied in 15 g single-use aluminium foil sachets that were stored at room temperature. Subjects were instructed to mix and dissolve the contents of the sachet in 200 mL of water and consume within 5 min.

Further blood draws were taken at weeks 4 and 8 for fasting glucose, fructosamine, and HbA1c determination, and at week 12 for the same with the addition of OGTT as described above.

### 2.7. Statistics

For in vitro experiments, significant differences from untreated controls were determined by one-way ANOVA followed by a Dunnett’s test. Data are presented as a percentage of untreated controls (mean ± SEM of at least three independent experiments). All statistical analyses were performed using the statistical computing software R [23].

For preclinical data, mean change from baseline was determined for each time point and expressed as the mean ± SEM for each treatment group. Total area under the curve (AUC) was calculated using the trapezoidal rule and treatment groups were compared by Student’s *t*-test. Additionally, for each time point, treatment groups were compared by ANOVA followed by Tukey’s test. For all analyses, a *p*-value < 0.05 was considered significant. Graphs were generated using GraphPad Prism version 8.0.0 for Windows, GraphPad Software, San Diego, CA, USA, www.graphpad.com (accessed on 7 May 2021).

For clinical data, two-way ANOVA or confounder analysis was utilised. For confounder analysis, general equating estimation (GEE) in a stepwise fashion in a participant-paired design was used. In the analysis, in addition to treatment, days of consumption, and their interaction, various confounding factors were taken into account, such as centre, age, gender, baseline values of diastolic blood pressure (DBP), respiration, pulse, temperature, and BMI. Outlier analysis was performed using Grubbs test. A *p*-value of 0.05 was considered to identify significance using two-sided evaluation. STATA, version 12.2 (StatCorp, College Station, TX, USA) was used for statistical evaluation and GraphPad Prism version 8.0.0 for Windows (GraphPad Software, San Diego, CA, USA, www.graphpad.com (accessed on 7 May 2021)) was used for graphical display.

## 3. Results

### 3.1. NRT_N0G5IJ Improves Glucose Uptake in Human Skeletal Muscle Cells

**Figure 1:**
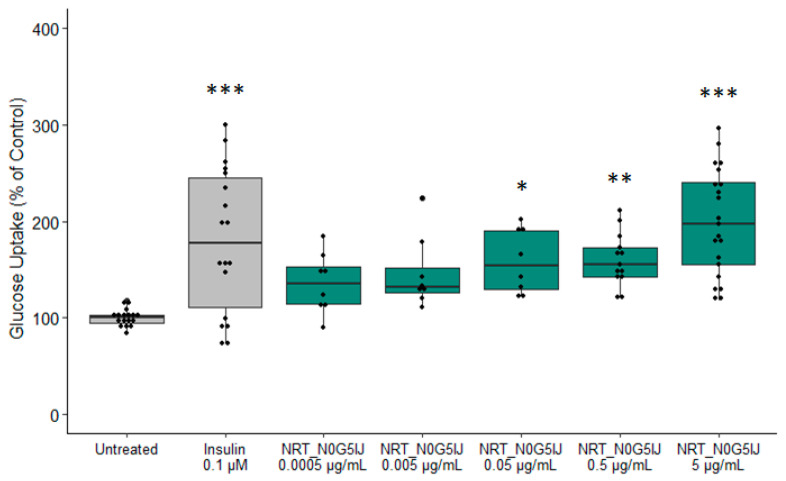
NRT_N0G5IJ significantly increases glucose uptake in human skeletal cells. Skeletal muscle cells were stimulated for 20 min with insulin (0.1 µM) or a dose of NRT_N0G5IJ (as indicated) prior to glucose uptake assessment with a minimum of three independent replicates (* *p* < 0.05; ** *p* < 0.01; *** *p* < 0.001).

To determine blood glucose uptake activity within the predicted *P. sativum* proteome, a functional ingredient, NRT_N0G5IJ, was produced and validated in vitro. Initial screening assessed the efficacy of NRT_N0G5IJ on glucose uptake. Glucose uptake was measured using 2-deoxyglucose (2-DG). 2-DG is taken up by glucose transporters and metabolised to 2-DG-6-phosphate (2-DG6P) which cannot be further metabolised by cells. The quantity of accumulated 2-DG6P is directly proportional to the amount of glucose uptake by cells. Following stimulation of the skeletal muscle cells, NRT_N0G5IJ significantly increased glucose uptake at higher concentrations compared to untreated skeletal muscle cells. (Figure 1). The 0.05 µg/mL (*N* = 3; *p* = 0.0237) and 0.5 µg/mL (*N* = 5; *p* = 0.0045) treatment groups displayed similar mean glucose uptake percentage; however, due to increased sample size and smaller error bars, a higher concentration of NRT_N0G5IJ resulted in a greater *p*-value. The 5 µg/mL NRT_N0G5IJ (*N* = 7; *p* < 0.0001) treatment resulted in glucose uptake similar to the positive control treatment group (insulin; (*N* = 7; *p* < 0.0001). Of note, unhydrolysed raw material did not elicit any significant effects on glucose uptake (Figure S1, Supplementary Materials).

**Figure 2:**
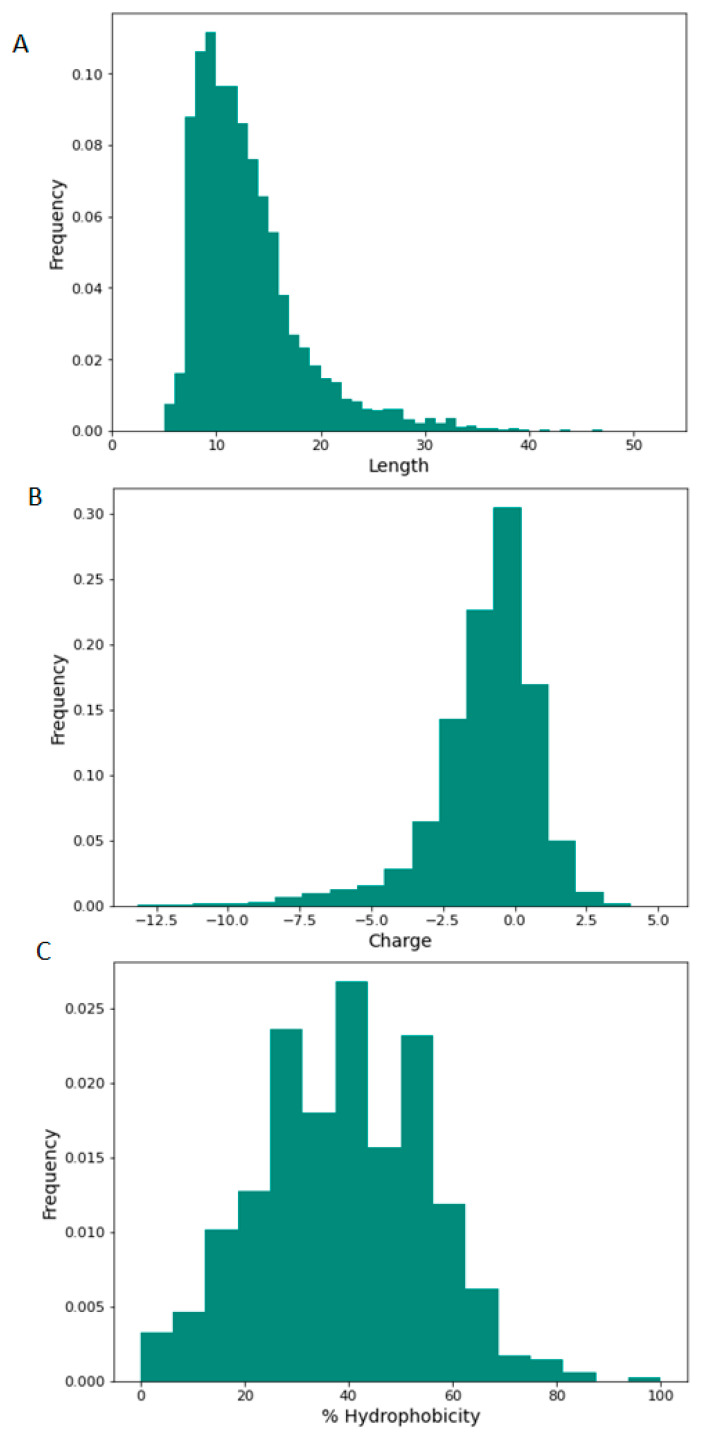
Physicochemical properties of NRT_N0G5IJ peptide profile as determined by LC–MS/MS. Histogram representation of NRT_N0G5IJ peptide distribution according to (**A**) length, (**B**) charge, and (**C**) percentage hydrophobicity; peptide counts are displayed on the *y*-axis.

We used LC–MS/MS to characterise NRT_N0G5IJ.The physiochemical properties of the peptides contained within NRT_N0G5IJ including sequence length, charge of peptides, and relative hydrophobicity are shown in Figure 2. The majority of NRT_N0G5IJ constituent peptides fall between seven and 16 amino acids in length (Figure 2A) and feature a global charge range from −4 to +1, where a net charge of +1 occurs most frequently (Figure 2B). Lastly, most of the peptides within NRT_N0G5IJ contain approximately 40% of hydrophobic residues (Figure 2C).

### 3.2. NRT_N0G5IJ Normalises Blood Glucose Regulation and Decreases HbA1c Concentrations in Diabetic Mice

**Figure 3:**
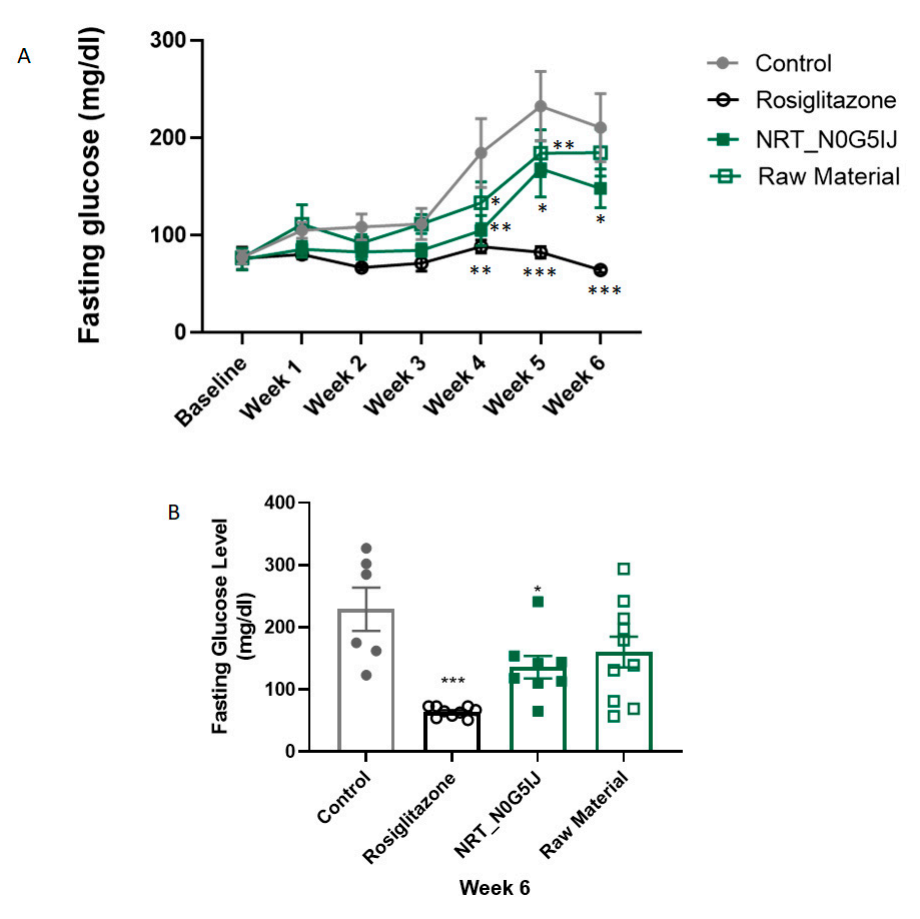
NRT_N0G5IJ significantly increases the ability to regulate blood glucose levels in db/db mice. Mice were treated with NRT_N0G5IJ (400 mg/kg/day), raw material (400 mg/kg/day), rosiglitazone (15 mg/kg/day), or vehicle by oral gavage. (**A**) Fasting blood glucose concentrations were determined weekly for 6 weeks. Two-way ANOVA analysis with Dunnett’s post hoc test. (**B**)) One-way ANOVA analysis of fasting blood glucose levels at week 6. Data are the mean ± SEM of *n* = 9 per group (* *p* < 0.05, ** *p* < 0.01; *** *p* < 0.001).

In an exploratory study, NRT_N0G5IJ, was orally administered daily to db/db mice for 43 days. The fasting glucose levels of all mice were measured weekly. At week 4, the fasting glucose level of mice treated with NRT_N0G5IJ was significantly lower than that of the vehicle control (Figure 3A), demonstrating an improved and sustained reduction in blood glucose. Although this effect was somewhat decreased at week 5 and 6 compared to week 4, there was a significant improvement over that observed with the vehicle control at the termination of this project (Figure 3B). As expected, pharmacological intervention with a small molecule, rosiglitazone, normalised glucose regulation. However, rosiglitazone is only useful as an experimental positive control as there have been significant side-effects reported that led to its withdrawal from market, again emphasising the need for safer alternatives [24]. Raw material did not result in any significant effects on FPG at any timepoint during treatment (Figure 3).

**Figure 4:**
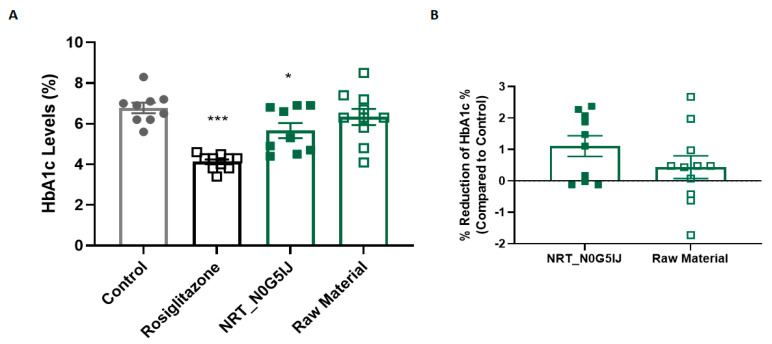
NRT_N0G5IJ significantly decreased HbA1c concentrations in diabetic db/db mice in a hydrolytic-specific manner. (**A**) The effect of rosiglitazone (15 mg/kg/day), NRT_N0G5IJ (400 mg/kg/day), or raw material (400 mg/kg/day) versus vehicle control on HbA1c concentrations in db/db mice was determined after 4 weeks of treatment. Mice were treated with 400 mg/kg/day of NRT_N0G5IJ, or vehicle by oral gavage. Data are the mean ± SEM (*n* = 9) after 4 weeks of treatment. (**B**) The effect of NRT_N0G5IJ and associated unhydrolysed raw material on HbA1c in db/db mice relative to untreated control mice was determined. Mice were treated with 400 mg/kg/day of NRT_N0G5IJ or raw material by oral gavage, or a similar volume (5 mL/kg) of vehicle. Data are the mean ± SEM (*n* = 9) after 4 weeks of treatment (* *p* < 0.05, *** *p* < 0.001).

HbA1c is a well-established and reliable measure for glucose metabolism and represents the average blood glucose level in an individual [25]. The effect of oral administration of NRT_N0G5IJ on HbA1c concentrations was characterised in vivo using db/db mice. Baseline HbA1c percentage levels of mice were not recorded; however, after 4 weeks, NRT_N0G5IJ-treated mice exhibited significantly reduced HbA1c concentrations to vehicle control mice, by 1.1% (Figure 4A). To confirm the importance of the hydrolysis process in releasing bioactive peptides, one group of db/db mice was treated with unhydrolysed raw material (*P. sativum*) that was used to produce NRT_N0G5IJ. Relative to the control, this unhydrolysed material showed no significant effect on HbA1c levels, whereas NRT_N0G5IJ showed a significant mean reduction of 1.1%, with a substantial, but not significant mean difference of 0.9% from the unhydrolysed material (Figure 4B).

**Figure 5:**
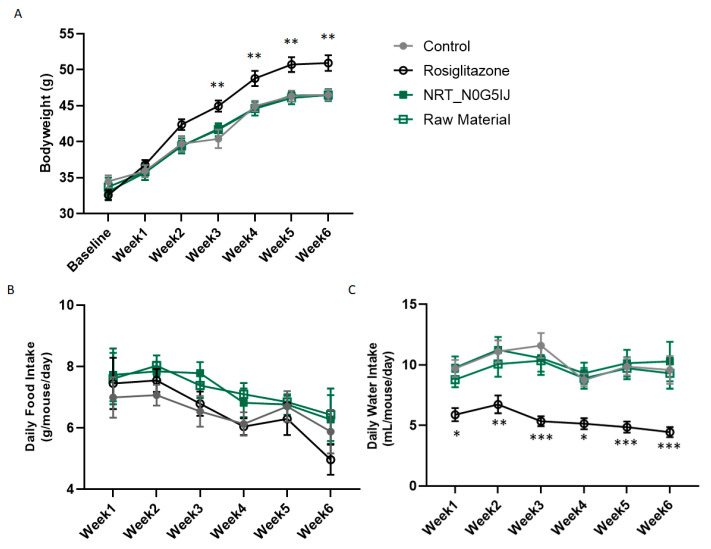
Effect of treatment on body weight, food, and water intake. Measurements of (**A**) body weight, (**B**) food intake, and (**C**) water intake on mice treated with NRT_N0G5IJ (400 mg/kg/day), raw material (400 mg/kg/day), rosiglitazone (15 mg/kg/day), or vehicle by oral gavage. Data are the mean ± SEM and analysed by Dunnett’s multiple comparisons tests as applicable (* *p* < 0.05, ** *p* < 0.01, *** *p* < 0.001; compared to vehicle).

Body weights of all mice were recorded weekly. Control mice gained weight as expected for this strain over time and a similar increase in body weight was observed in mice treated with NRT_N0G5IJ. However, mice treated with rosiglitazone showed significantly increased body weights (Figure 5A). Food intake was comparable across all treatment cohorts (Figure 5B), but water intake was significantly reduced in mice treated with rosiglitazone (Figure 5C).

### 3.3. NRT_N0G5IJ Decreases HbA1c in Humans

**Figure 6:**
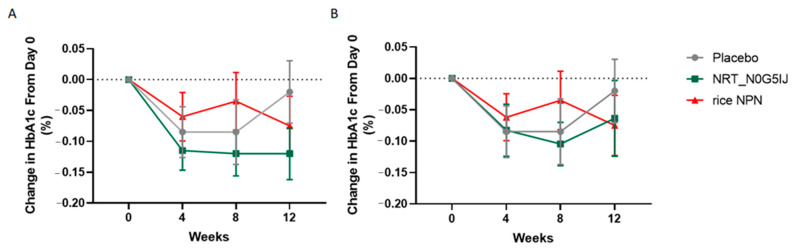
NRT_N0G5IJ treatment decreased HbA1c percentage in a prediabetic population. NRT_N0G5IJ (15 g/day) administration elicited a significant decrease in HbA1c relative to either placebo or control rice NPN in (**A**) per protocol (PP) or (**B**) intention to treat (ITT) populations (GEE analysis; data are represented as the mean ± SEM).

In order to assess the effect of NRT_N0G5IJ in a human population, subjects were screened for HbA1c concentrations and randomly assigned to one of three treatment arms: (i) placebo, (ii) NRT_N0G5IJ, or (iii) a peptide network hydrolysed from rice (rice NPN) as a protein control. Over 12 weeks of treatment, a decrease of 0.12% was observed in HbA1c percentage (Figure 6). After Grubbs’ test analysis, two outliers were removed from the NRT_N0G5IJ group on the basis of “interaction”, which was defined as the time-dependent change in percentages between groups. Figure 6A details the change in HbA1c percentage over 12 weeks in the per protocol (PP) population; here, we see a significant reduction in HbA1c percentage following NRT_N0G5IJ treatment compared to control (*N* = 20; *p* = 0.013). No interaction was detected between placebo (*N* = 20) and rice NPN (*N* = 20), while there were also no interactions observed between NRT_N0G5IJ and rice NPN cohorts. When examining the ITT population, a significant reduction in HbA1c percentage was still observed with NRT_N0G5IJ (*N* = 23) treatment compared to placebo treatment (*p* = 0.05; Figure 3B). Those supplemented with NRT_N0G5IJ exhibited significantly greater reduction in HbA1c percentage compared to placebo (*N* = 21) or rice NPN (*N* = 21) cohorts, indicating that the effect of peptide network on HbA1c percentage was protein-dependent. In this study, changes in secondary outcomes (baseline values supplied in Table S1, Supplementary Materials), such as fasting glucose (Figure S2, Supplementary Materials), change in BMI (Figure S3, Supplementary Materials),OGTT (Figure S4, Supplementary Materials), or insulin levels (Figure S5, Supplementary Materials), were not observed during the time frame of the study for any cohort. There was no effect on weight in either treatment group compared to placebo; however, there was a significantly slower weight increase observed in the rice NPN cohort compared to NRT_N0G5IJ (Figure S6, Supplementary Materials; PP, *p* = 0.021; ITT, *p* = 0.036). An additional interaction was observed between NRT_N0G5IJ and rice NPN treatment regarding fructosamine levels. Following 12 weeks of treatment, NRT_N0G5IJ significantly reduced fructosamine levels compared to rice NPN (Figure S7, Supplementary Materials; PP, *p* = 0.002; ITT, 0.015), although neither cohort was significantly different from placebo.

Adverse events (AE) were reported for 11.1% subjects in the rice NPN group, 16.0% in the NRT_N0G5IJ group, and 8.0% in the placebo group. There were no subjects with serious AEs (SAE) reported in the rice NPN group and NRT_N0G5IJ group. There was one subject with SAE, which occurred in the placebo group, who experienced segmental colitis of sigmoid colon which was possibly related to study treatment. The subject stopped the treatment (discontinued). Adverse events considered moderate were observed for three subjects: one subject in the placebo group and two subjects in the rice NPN group. However, most of the adverse events were mild in severity and resolved without treatment. Overall, the study indicates that rice NPN (15 g) and NRT_N0G5IJ were (15 g) safe and well tolerated, similar to placebo.

## 4. Discussion

In this study, we aimed to utilise an AI approach to discover a functional ingredient capable of modulating glucose levels. Following prediction of *P. sativum* as an optimal plant source for a network of bioactive peptides with glucose metabolism activity, NRT_N0G5IJ was produced and validated in vitro for activity and safety. This was followed by a diabetic murine model trial which confirmed efficacy and culminated in a proof-of-concept human trial carried out in a relevant population.

Bioactive peptide discovery has been accelerated through the use of AI and machine learning methods, with the resulting identification of functional ingredients derived from different nutritional sources such as rice and fava bean [18,20]. While different functional foods have been tested for their ability to regulate blood glucose and initial data suggest this could be a promising nonmedical intervention [26], members of the Fabaceae family, of which *P. sativum*, is a member, have been linked to glucose regulation [27]; however, we observed that treatment with the unhydrolysed source material only did not exhibit any significant effects on glucose uptake. These results highlight the importance of the manufacturing process, and, as digestion is different across individuals, it is probable that the bioactive peptides will not be released consistently across the population.

Db/db mice with a C57BLKS/J genetic background are a well-studied mouse model for type 2 diabetes [28], and they develop morbid obesity, chronic hyperglycaemia, and pancreatic beta cell atrophy over time [29]. As bioavailability of NRT_N0G5IJ was not evaluated at the time of this exploratory study design, a high concentration was utilised; however, further work is currently underway to determine bioaccessibility and stability of NRT_N0G5IJ to improve dose optimisation. We observed a significant improvement in FPG levels, and a significant decrease in HbA1c was observed compared to placebo, following daily administration of NRT_N0G5IJ. Raw material supplementation had no effect on FPG at the termination of this study; additionally, no effect on HbA1c percentage was recorded. These findings clearly indicate the HbA1c percentage reduction is dependent upon the specific network of peptides present in NRT_N0G5IJ, highlighting the importance of unlocking bioactive peptides from plant-based sources prior to consumption. Similarly, lack of activity in the raw material was described in a study where effect on muscle protein synthesis was specific to unlocked bioactive peptides, with no effect being observed following raw material treatment [20]. Additionally, food intake was not effected by any treatment; however, mice receiving rosiglitazone exhibited significantly decreased water intake, which was previously reported in a similar murine model [30]. Weight of NRT_N0G5IJ-treated mice was comparable to controls; however, rosiglitazone treatment significantly increased body weight, despite slightly lower recorded food intake. Of note, rosiglitazone treatment has been previously shown to increase body weight and adiposity in ob/ob mice, although a significant increase in food intake was also observed; this difference may be mouse strain-specific [31]. Moreover, increase in body weight was a common effect observed in previous clinical studies with various antidiabetic drugs, and this not only has proven to be a barrier in achieving sufficient glycaemic control but it can also potentially lead to other problems associated with increased weight [32]. Therefore, NRT_N0G5IJ offers a promising alternative to glucose level regulation without the possible adverse effect of body weight gain.

Following a 12 week double blinded placebo trial with daily administration of NRT-N0G5IJ, a small, yet significant, decrease in HbA1c percentage was observed in a prediabetic population. These results were not observed in the placebo cohort or a protein hydrolysate control cohort which received a rice-derived hydrolysate with validated anti-inflammatory effects in an aging population in vivo. Interestingly, Stratton et al. (2000) showed that, for each 1% reduction in HbA1c, there was an associated reduction in risk of 21% for any end point related to diabetes, 14% for myocardial infarction, and 37% for microvascular complications [33]. The results achieved in this present study are potentially noteworthy as a previous study, the EPIC-Norfolk population study, assessed the estimated effect of diabetes incidence distribution if HbA1c levels were lowered. It was predicted that lowering HbA1c concentration by 0.1%, similar to HbA1c reduction in the current trial, would lead to an estimated prevention of 12% of excess deaths [34]. The study also showed that reducing HbA1c levels by 0.1–0.2% reduced the incidence of pre-diabetes from 79% to 63% and 57% respectively in the general population. Additionally, these reductions in prevalence would reduce the mortality rate in the general population by 5–10% [35]. Conversely, an increase of even 1% in HbA1c level has been linked with an increased risk of mortality by 28%, and this would occur in the general population, regardless of age or any other influential aspect [35].

Taking these results into consideration, it appears likely that the mean HbA1c reduction of −0.12% observed in this study could potentially elicit health benefits in a prediabetic population. The study duration is also critical as NRT_N0G5IJ elicited a change in HbA1c after 12 weeks of dietary supplementation. Current guidelines suggest testing every 6 months [8], while some studies show that annual testing would suffice to detect changes [36]. Further applications of NRT_N0G5IJ may be indicated as T2DM patients with stable glycaemic control increase their HbA1c by 0.27% (3 mmol/mol) every year [36], potentially showing that not only would diabetic or prediabetic patients benefit from NRT_N0G5IJ, but it could also be applicable to the ageing population maintaining HbA1c levels.

Despite the significant reduction in HbA1c percentage, postprandial glucose/insulin levels were not impacted by NRT_N0G5IJ treatment. HbA1c is considered a more robust predictor for development of T2DM than fasting plasma glucose (FPG) and oral glucose tolerance test (OGTT) due to higher test sensitivity. Alqahtani et al. previously reported that FPG and OGTT failed to detect diabetes in 56% and 37.5% of cases [37]. Of note, while not recorded in this study, rice NPN was previously shown to have a mild effect on OGTT in an inflammaging population [18]; this effect was observed following a substantially higher dose and increased frequency of administration, indicating the importance of dose optimisation in the validation of functional ingredients, with further investigation warranted.

Management of prediabetes focuses on the prevention of diabetes development and the subsequent health and financial consequences of diabetes. A large proportion of the published literature suggests that lifestyle interventions are key for the control of progression to diabetes [21]. This approach involves focusing on dietary changes and increased physical activity [38]. The Mediterranean and plant-based diets, in particular, have been shown to be effective in improving glycaemic control and reducing the risk for T2DM [39,40,41,42]. However, compliance with these diets can make them difficult to implement as a long-term strategy. Two of the largest diabetes prevention studies, showing the benefits of lifestyle interventions, include the Finnish Diabetes Prevention study (DPS) and the United States DPP [43]. For the DPP study, there was a 58% risk reduction identified at 3 years follow-up, following intensive lifestyle interventions (ILS) [44]. Outside of lifestyle intervention, the only current intervention pharmaceutical strategies include therapeutic options such as biguanides, thiazolidinediones, antiobesity drugs, and surgery, among others, with metformin being the gold-standard drug for treatment of T2DM [45]. Interestingly, DPP data have indicated lifestyle interventions to be as effective as small-molecule therapeutics [43].

These data, coupled with low compliance rates for lifestyle interventions, the ever-growing prevalence of diseases such as diabetes and cardiovascular disease, and an innate dependency on pharmaceuticals, position orally delivered protein-derived peptide networks as attractive nutritional prevention strategies. This is also supported by safe toxicity profiles of peptides and functional ingredients [46,47,48]. Of relevance, in this study, we saw a significant reduction in HbA1c despite diet and physical activity not being controlled, with participants being asked to maintain their usual diet and physical activity levels for the duration of the study.

## 5. Conclusions

The number of individuals with prediabetes is increasing rapidly, as is the global incidence of type 2 diabetes, currently affecting 425 million people, and this is expected to increase by a further 204 million by 2045, indicating an opportunity for a functional ingredient to maintain healthy glucose levels. To develop a validated functional ingredient, we employed an AI strategy for discovery. Following the identification of NRT_N0G5IJ and initial assessment in vitro, we observed a significant benefit on HbA1c in mice, which translated to a human clinical trial setting. This effect was achieved after just 12 weeks of daily administration of NRT_N0G5IJ. Although the effects were modest and a further long-term study and dose optimisation are warranted, these initial significant changes in HbA1c concentrations could result in concomitant significant health benefits.

## Figures and Tables

**Table 1:** Subject demographic for trial NCT03851666.

	Placebo	Rice NPN	NRT_N0G5IJ
Number (*n*)	25	27	25
Mean age (range, years)	58.8 (33–75)	59.3 (33–75)	55.8 (26–75)
Male (*n*, %)	10 (40%)	8 (29.6%)	11 (44%)
Female (*n*, %)	15 (60%)	19 (70.4%)	14 (56%)
Mean height (range, cm)	170.58 (152–188)	167.93 (155–200)	171.40 (155–190)
Mean weight (range, kg)	90.61 (70–115.3)	90.12 (64.6–129.5)	88.82 (59.5–123)
Mean BMI (range, kg/m^2^)	31.14 (24.5–34.95)	31.77 (23.7–34.9)	30.04 (20.8–34.78)
Mean HbA1c (range, %)	5.99 (5.7–6.4)	6.04 (5.7–6.3)	6.04 (5.7–6.2)

## Data Availability

The data presented in this study are available on request from the corresponding author. The data are not publicly available due to IP protection, including protection of know-how and inventions not yet subject to patent protection.

## Supplementary Materials

The following are available online at https://www.mdpi.com/article/10.3390/nu13051635/s1: Figure S1. Effect of raw material on glucose uptake in human skeletal cells; Table S1. Baseline values for trial NCT03851666; Figure S2. Effect of NRT_N0G5IJ on fasting glucose; Figure S3. Effect of NRT_N0G5IJ on BMI; Figure S4. Effect of NRT_N0G5IJ on OGTT; Figure S5. Effect of NRT_N0G5IJ on Insulin; Figure S6. Effect of NRT_N0G5IJ on weight; Figure S7. Effect of NRT_N0G5IJ on fructosamine.
