# Supplementary material for: An Artificial-Intelligence-Discovered Functional Ingredient, NRT_N0G5IJ, Derived from Pisum sativum, Decreases HbA1c in a Prediabetic Population
Source: Nutrients. 2021 May 13;13(5):1635. doi: 10.3390/nu13051635 (PMC8152294; doi:10.3390/nu13051635)
Supplement: Supplementary file 1 [file nutrients-13-01635-s001.zip › nutrients-1186778-supplementary.pdf]

nutrients

MDPI

Supplementary

An Artificial Intelligence Discovered Functional Ingredient, NRT_N0G5IJ, Derived from *Pisum sativum*, Decreases HbA1c in a Prediabetic Population.

Sweeny Chauhan, Alish Kerr, Brian Keogh, Stephanie Nolan, Rory Casey, Alessandro Adelfio, Niall Murphy, Aoife Doherty, Heidi Davis, Audrey M. Wall * and Nora Khaldi

Table S1. Baseline values for trial NCT03851666.

	Placebo	Rice NPN	NRT_N0G5IJ
Fasting Glucose, mmols/L<br>Mean (SD)<br>*Range*	6.003 (0.7672)<br>*5.07–8.07*	5.702 (0.6538)<br>*4.62–7.34*	5.656 (0.7141)<br>*4.54–7.85*
Weight, kg<br>Mean (SD)<br>*Range*	90.61 (10.86)<br>*70–115.3*	90.12 (16.08)<br>*64.6–129.5*	88.82 (17.18)<br>*59.5–123*
BMI, kg/m^2<br>Mean (SD)<br>*Range*	31.14 (3.35)<br>*24.5–34.95*	31.77 (3.32)<br>*23.7–34.9*	30.04 (4.22)<br>*20.8–34.78*
Fructosamine , micromol/L<br>Mean (SD)<br>*Range*	243.6 (25.64)<br>*152–262*	254.9 (16.38)<br>*219–294*	243.2 (20.28)<br>*206–276*
OGTT, mmol/L<br>Mean (SD) AUC values for Baseline	18.138 (4.3369)	17.980 (3.9801)	16.746 (3.1087)
Insulin, microU/mL<br>Mean (SD) AUC values for Baseline	168.06 (102.970)	136.73 (120.724)	212.06 (161.076)

Nutrients **2021**, *13*, 1635. https://doi.org/10.3390/nu13051635 https://www.mdpi.com/journal/nutrients

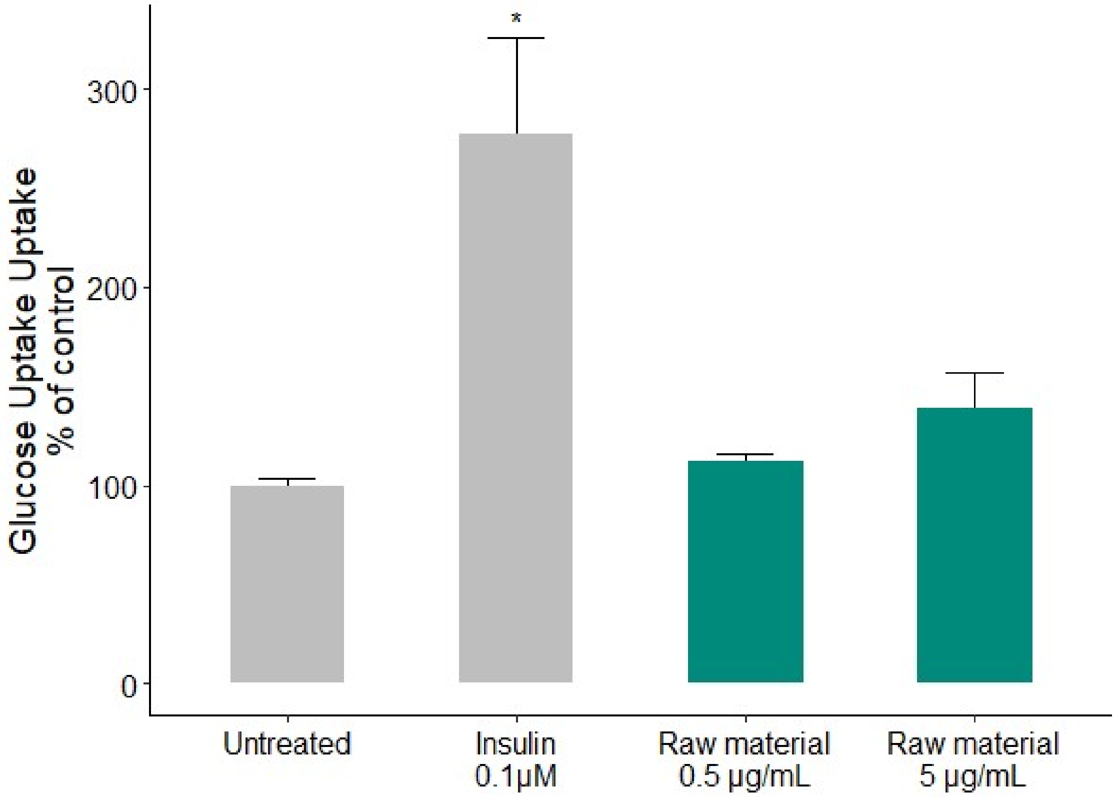

Figure S1. Effect of raw material on glucose uptake in human skeletal cells. Skeletal muscle cells were stimulated for 20 minutes with Insulin (0.1µM) or a dose of raw material (as indicated) prior to glucose uptake assessment. (Data is represented as mean ± SD; * $p < 0.05$).

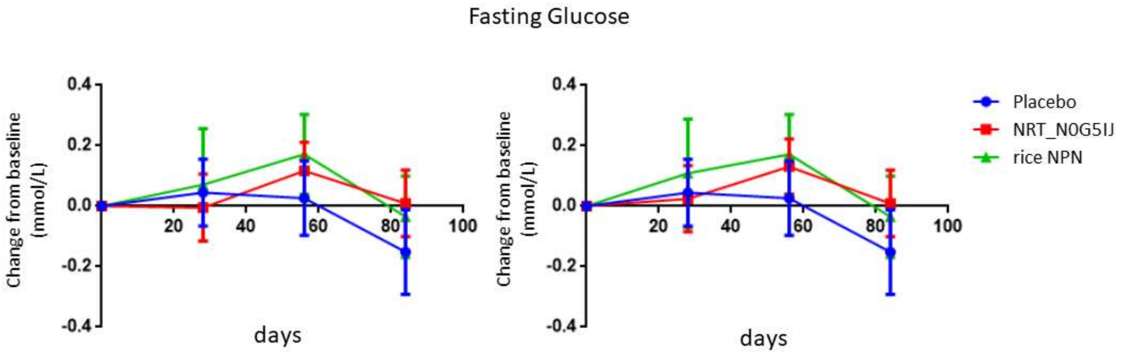

(A) **(B)**

Figure S2. Effect of NRT_N0G5IJ on fasting glucose. Change in fasting glucose during 12 weeks of consumption of either NRT_N0G5IJ, rice NPN or placebo from baseline values. Each symbol represents the mean +/- 1 x S.E.M. of at least 18 observations in **A)** per protocol and **B)** intention to treat populations.

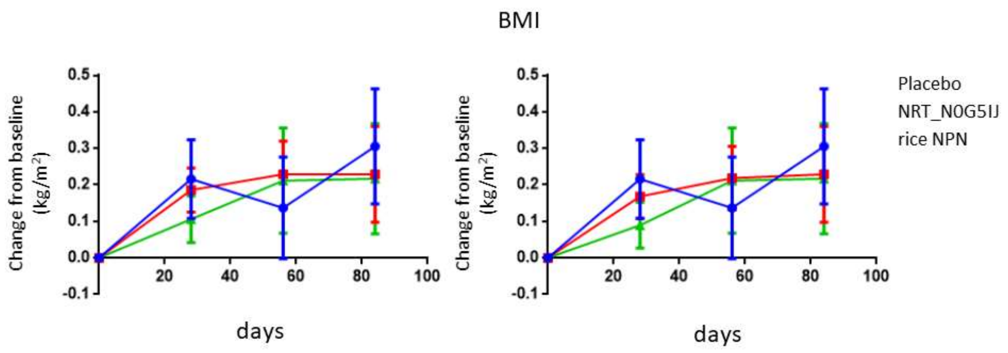

(A) **(B)**

Figure S3. Effect of NRT_N0G5IJ on BMI. Change in BMI during 12 weeks of consumption of either NRT_N0G5IJ, rice NPN or placebo. Outliers have been removed. Each symbol represents the mean +/- 1 x S.E.M. of at least 18 observations in **A)** per protocol and **B)** intention to treat populations.

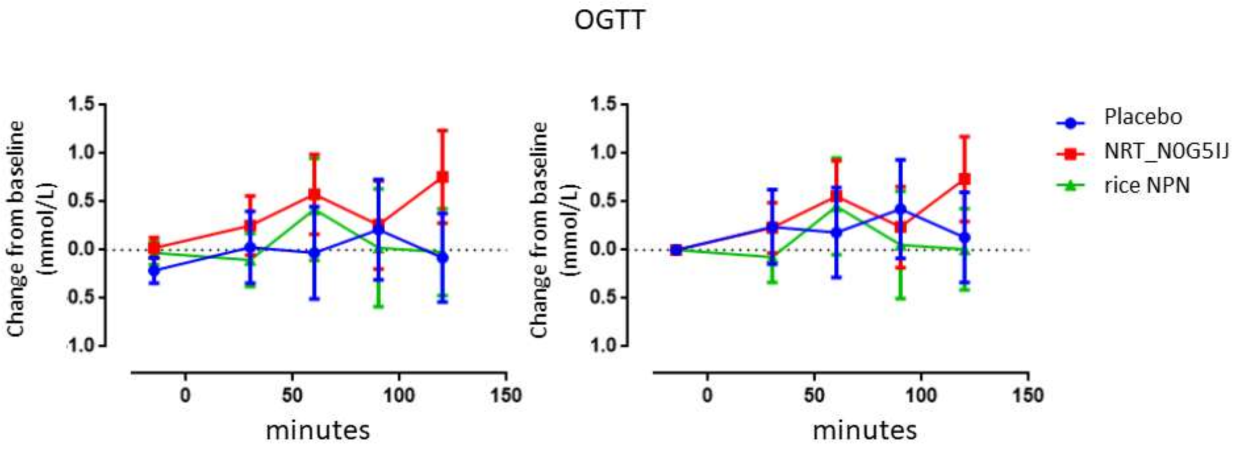

(A) **(B)**

Figure S4. Effect of NRT_N0G5IJ on OGTT. Change in OGTT values over 12 weeks of consumption of either NRT_N0G5IJ, rice NPN or placebo in the intention to treat population where **A)** Outliers have been removed and **B)** outliers have not been removed. The per protocol population yielded similar results. Each symbol represents the mean +/- 1 x S.E.M. of at least 19 observations.

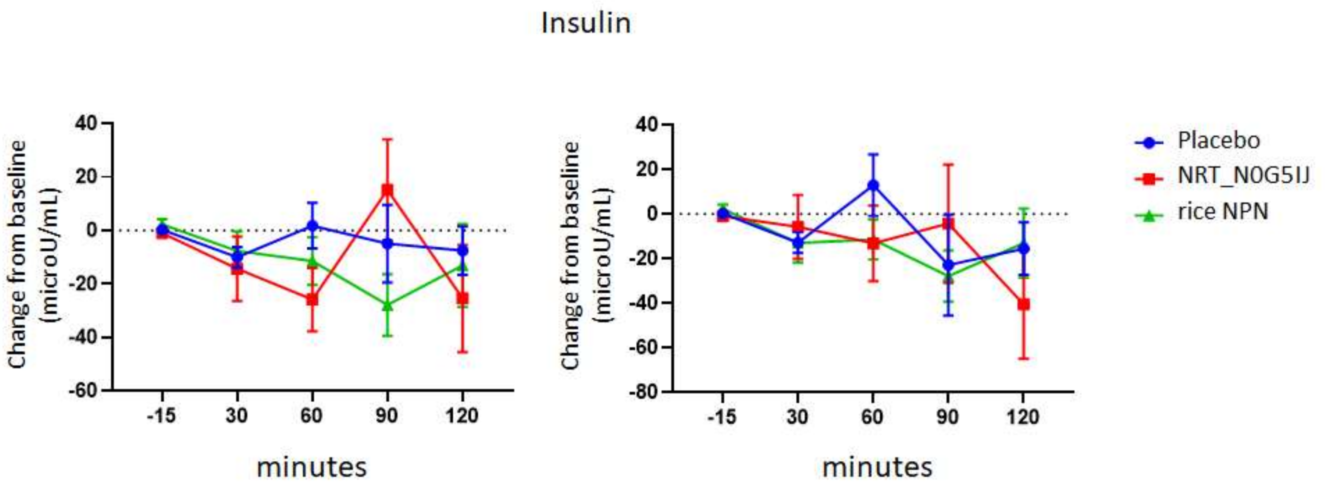

(A) **(B)**

Figure S5. Effect of NRT_N0G5IJ on Insulin. Change in insulin values over 12 weeks of consumption of either NRT_N0G5IJ, rice NPN or placebo in the intention to treat population where **A)** Outliers have been removed and **B)** outliers have not been removed. The per protocol population yielded similar results. Each symbol represents the mean +/- 1 x S.E.M. of at least 19 observations.

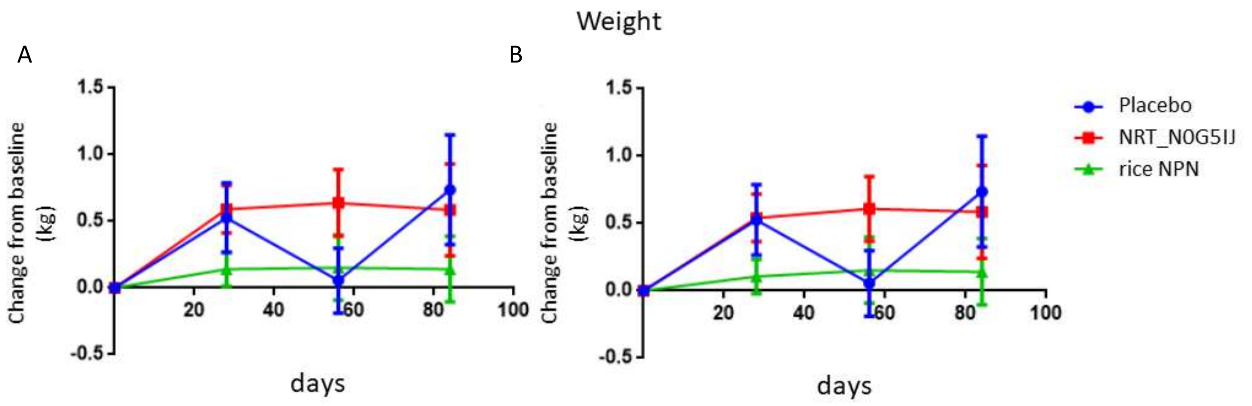

(A) **(B)**

Figure S6. Effect of NRT_N0G5IJ on weight. Change in weight during 12 weeks of consumption of either NRT_N0G5IJ, rice NPN or placebo. Outliers have been removed. Each symbol represents the mean +/- 1 x S.E.M. of at least 17 observations in **A)** per protocol and **B)** intention to treat populations.

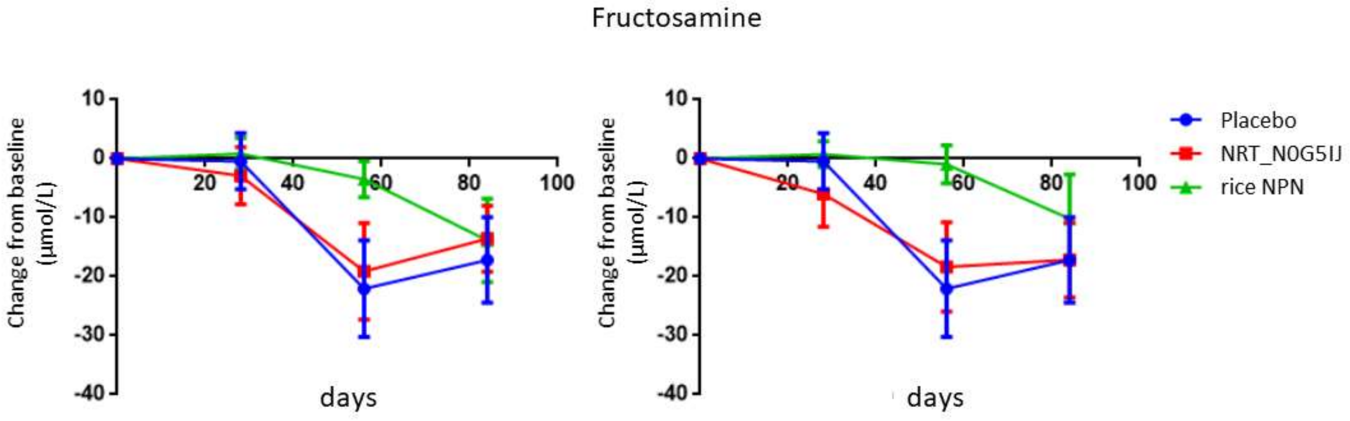

(A) **(B)**

Figure S7. Effect of NRT_N0G5IJ on fructosamine. Change in fructosamine during 12 weeks of consumption of either NRT_N0G5IJ, rice NPN or placebo. Outliers have been removed. Each symbol represents the mean +/- 1 x S.E.M. of at least; 13 observations in **A)** per protocol; and **B)** 15 observations in intention to treat populations.
